# Heterozygous *RHO* p.R135W missense mutation in a large Han-Chinese family with retinitis pigmentosa and different refractive errors

**DOI:** 10.1042/BSR20182198

**Published:** 2019-07-12

**Authors:** Yuan Wu, Yi Guo, Junhui Yi, Hongbo Xu, Lamei Yuan, Zhijian Yang, Hao Deng

**Affiliations:** 1 Center for Experimental Medicine and Department of Neurology, The Third Xiangya Hospital, Central South University, Changsha, China; 2 Department of Clinical Laboratory, The Third Xiangya Hospital, Central South University, Changsha, China; 3 Department of Medical Information, School of Life Sciences, Central South University, Changsha, China; 4 Department of Ophthalmology, The Third Xiangya Hospital, Central South University, Changsha, China

**Keywords:** missense mutation, retinitis pigmentosa, rhodopsin, whole exome sequencing

## Abstract

Retinitis pigmentosa (RP), the most common type of inherited retinal degeneration causing blindness, initially manifests as severely impaired rod function followed by deteriorating cone function. Mutations in the rhodopsin gene (*RHO*) are the most common cause of autosomal dominant RP (adRP). The present study aims to identify the disease-causing mutation in a numerous, four-generation Han-Chinese family with adRP detected by whole exome sequencing and Sanger sequencing. Afflicted family members present classic adRP along with heterogeneous clinical phenotypes including differing refractive errors, cataracts, astigmatism and epiretinal membranes. A missense mutation, c.403C>T (p.R135W), in the *RHO* gene was identified in nine subjects and it co-segregated with family members. The mutation is predicted to be disease-causing and results in rhodopsin protein abnormalities. The present study extends the genotype–phenotype relationship between *RHO* gene mutations and adRP clinical findings. The results have implications for familial genetic counseling, clinical management and developing RP target gene therapy strategies.

## Introduction

Retinitis pigmentosa (RP, OMIM 268000) is the most common type of inherited, blindness-causing retinal degeneration. Its prevalence is ∼0.020–0.033% in general populations with no apparent ethnic or geographic distinctions [1,2]. The patient initially suffered from seriously impaired rod function followed by impaired cone function. Clinical features include night blindness, visual field constriction, visual acuity impairment, fundus degeneration and late-life vision loss [1]. There are three primary Mendelian RP inheritance modes: autosomal dominant (30–40%), autosomal recessive (50–60%) and X-linked (5–15%). Mitochondrial, or digenic forms are rare but have been reported [1,3]. Most RP patients have myopic refractive errors, especially in X-linked RP [4–7]. To date, more than 79 genes have been reported as related to RP [1]. Mutations in genes including the rhodopsin gene (*RHO*), the pre-mRNA processing factor 31 gene (*PRPF31*), the RP GTPase regulator gene (*RPGR*), the peripherin 2 gene (*PRPH2*), the RP1 axonemal microtubule associated gene (*RP1*), the inosine monophosphate dehydrogenase 1 gene (*IMPDH1*), the pre-mRNA processing factor 8 gene (*PRPF8*) and the nuclear receptor subfamily 2 group E member 3 gene (*NR2E3*) can cause autosomal dominant RP (adRP), and each accounts for at least 2% of all patients [8,9]. *RHO* gene mutations are the most common cause of adRP and account for 16–35% of adRP cases in Western populations. The frequency is much lower (2.0–7.7%) in Chinese, Japanese, Korean and South Asian Indian patients, indicating that the frequency may be ethnicity dependent [10–12].

In the present study, whole exome sequencing (WES) and Sanger sequencing were performed to identify possible genetic causes for the presence of adRP in a four-generation Han-Chinese family. An *RHO* gene missense mutation, c.403C>T (p.R135W), was identified and found to co-segregate with the adRP phenotype, supporting the hypothesis that it is the genetic cause of the adRP.

## Methods

### Participators and clinical evaluation

**Figure 1:**
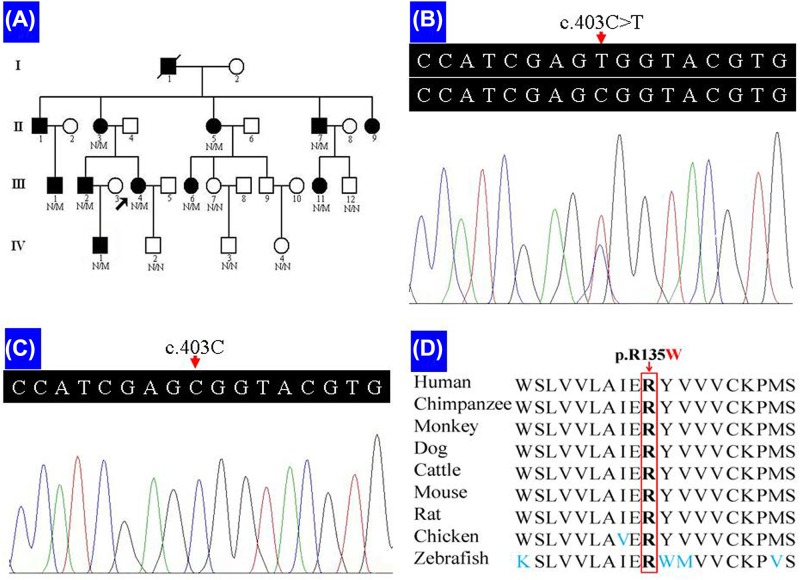
Pedigree data of an adRP family (**A**) Pedigree of the family with adRP. N: normal; M: the *RHO* c.403C>T (p.R135W) mutation. Arrow indicates the proband. (**B**) Sequence of heterozygous c.403C>T (p.R135W) mutation. (**C**) Sequence of a normal control. (**D**) Conservation analysis of the rhodopsin p.R135 amino acid residue.

A 27-person, four-generation Han-Chinese pedigree was enrolled at the Third Xiangya Hospital, Central South University, Changsha, Hunan, China (Figure 1A). Clinical data and peripheral blood samples were obtained from 14 family members, including nine affected (II:3, II:5, II:7, III:1, III:2, III:4, III:6, III:11 and IV:1) and five unaffected individuals (III:7, III:12, IV:2, IV:3 and IV:4). Blood samples were collected from 100 unrelated, ethnically matched normal controls (male/female: 50/50, age 38.5 ± 5.6 years). Informed written consent was obtained from each or their guardians. The study complied with the Declaration of Helsinki and was approved by the Ethics Committee of the Third Xiangya Hospital of Central South University in China. Participants underwent visual acuity testing by E decimal charts. Best corrected visual acuity (BCVA) was recorded as a logarithm of the minimum angle of resolution (logMAR) with cycloplegic refraction. Subjects were also examined and measured with slit-lamp biomicroscopy, fundus examination, full-field electroretinography and optical coherence tomography (OCT). A diagnosis of adRP was based on family history and typical clinical characters including presence of night blindness, reduced peripheral visual fields, typical fundus findings and abnormal OCT results [3].

### Variant genetic analysis and bioinformatics analysis

WES was performed by the Novogene Bioinformatics Institute, Beijing, China, using gDNA from the proband (III:4). After a DNA quality assessment, the captured DNA library was sequenced using a HiSeq 2000 platform to generate 150-bp paired-end reads as manufacturer protocols (Illumina Inc., San Diego, CA, U.S.A.) [13,14].

Analysis-ready BAM alignment data were obtained after duplicate removal, local alignment and base quality recalibration by Picard (http://sourceforge.net/projects/picard/), Genome Analysis Toolkit and SAMtools. All obtained variants were further screened using data from public variant databases including the Single Nucleotide Polymorphism Database (dbSNP build 137, http://www.ncbi.nlm.nih.gov/projects/SNP/snp_summary.cgi), 1000 genomes project, and the NHLBI Exome Sequencing Project (ESP) 6500 [15]. Only variants occurring in exon regions or canonical splicing sites, with a minor allele frequency of less than 0.01 were analyzed further. Sorting Intolerant from Tolerant (SIFT) and Polymorphism Phenotyping version 2 (PolyPhen-2) were used to predict functional effects of the nonsynonymous single nucleotide polymorphisms (SNPs) [16,17]. The candidate gene variant related to vision degeneration disease was prioritized for validation, as were those performed in recent studies [18–20]. Sanger sequencing was performed to confirm the potential pathogenic variant using ABI3500 sequencer (Applied Biosystems, Foster City, CA, U.S.A.) [18]. PCR amplification and Sanger sequencing primer sequences are as follows: 5′-CTCCTTAGGCAGTGGGGTCT-3′ and 5′-GGAGCTTCTTCCCTTCTGCT-3′.

Multiple sequence alignments among different species were performed using the NCBI Basic Local Alignment Search Tool (http://blast.st-va.ncbi.nlm.nih.gov/Blast.cgi). MutationTaster (http://www.mutationtaster.org/) was used to further assess amino acid alteration potential pathogenicity [20,21].

### Subcellular location prediction

The rhodopsin protein consists of cytoplasmic (CP), transmembrane (TM) an extracellular (EC) domains. TransMembrane prediction using Hidden Markov Models (TMHMM) Server v. 2.0 (http://www.cbs.dtu.dk/services/TMHMM/) was applied to predict TM helices in wild-type rhodopsin and the structural interpretation of mutated rhodopsin.

## Results

### Pedigree clinical characteristics

**Figure 2:**
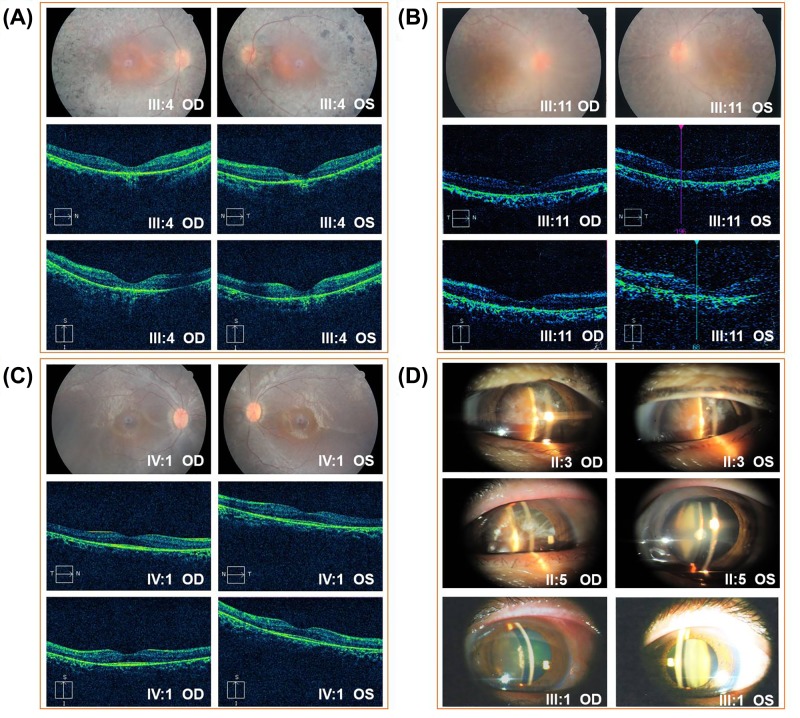
Fundus, OCT and slit lamp photographs of the patients (**A**–**C**) Fundus photographs and OCT images for patients (III:4, III:11 and IV:1). (**D**) Slit lamp photographs of patients (II:3, II:5 and III:1). Bilateral eyes of the patients (II:3 and II:5) and the left eye of the patient III:1 showed cataract, and the right eye of the patient III:1 presented with glaucoma secondary to traumatism.

**Figure 3:**
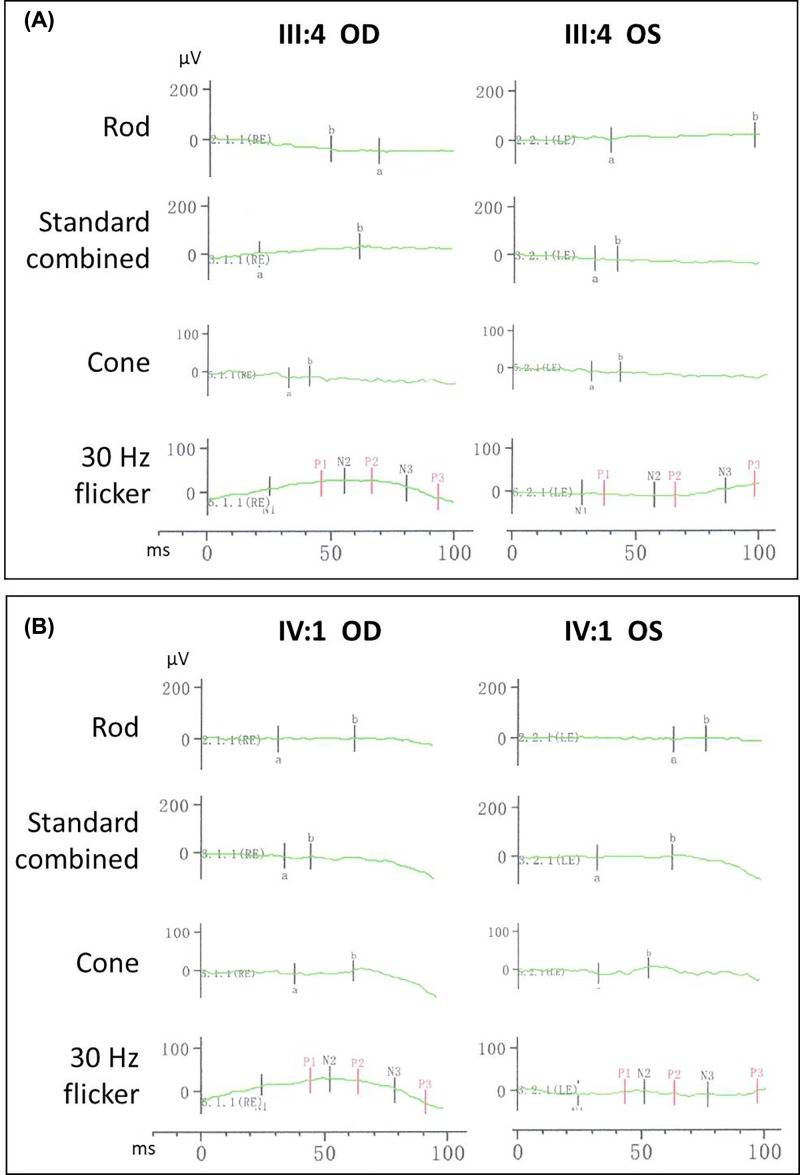
Electroretinography results for patients III:4 and IV:1 Non-recordable ERG results were observed in patients III:4 (**A**) and IV:1 (**B**).

Nine family members, four males and five females, were diagnosed with RP by two independent ophthalmologists. Phenotype transmission among four generations in this pedigree supports an autosomal dominant inheritance pattern (Figure 1A). All affected patients began suffering severe night blindness at 1–2 years, exhibited characteristic clinical symptoms including reduced periphery visual fields after 30 and ultimately developed blindness in later life (II:3 at 45; II:5 at 50; and II:7 at 45). Fundus examinations presented bone spicule-like pigmentation in the inferior periphery (Figure 2A,B), retinal pigment epithelium atrophy and retinal vessel attenuation (Figure 2A-C). OCT detected severely thinned (except III:2 and IV:1) and disorganized, inner and outer, photoreceptor segments (Figure 2A,B). Non-recordable ERG results were observed in patients III:4 and IV:1 (Figure 3). Intriguingly, patients III:2 and IV:1 had serious hyperopic refractive errors. Patients II:3 and II:5 had shorter axial lengths than healthy individuals, indicating hyperopic refractive errors. Patients III:1, III:4 and III:6 have myopic refractive errors. Except for a 9-year-old boy (IV:1), all patients present with, moderate or severe, cataracts (Figure 2D). One patient (III:2) has an epiretinal membrane (EM) and six patients (III:1, III:2, III:4, III:6, III:11 and IV:1) have astigmatism. The clinical and genetic data for nine patients are summarized in Table 1.

**Table 1:** Clinical and genetic data of nine patients with *RHO* c.403C>T (p.R135W) variant

Subject	II:3	II:5	II:7	III:1	III:2	III:4	III:6	III:11	IV:1
Age (years) /Sex	62/F	60/F	56/M	39/M	42/M	41/F	35/F	32/F	9/M
Onset age (years)	2	2	2	2	2	1	2	1	2
Genotype	Heterozygote	Heterozygote	Heterozygote	Heterozygote	Heterozygote	Heterozygote	Heterozygote	Heterozygote	Heterozygote
BCVA (OD/OS)	LP (5 m/5 m)	LP (5 m/5 m)	NLP/NLP	NLP/0.1	0.3/0.3	0.1/0.04	0.2/0.2	0.04/0.06	0.5/0.4
Optometry (OD)	NA	NA	NA	NA	+5.50/+1.50 × 30°	−0.75×90°	−5.00/−2.00×180°	+0.50/−3.00×180°	+3.00×105°
Optometry (OS)	NA	NA	NA	−9.5/−2.0 × 165°	+4.00	−1.75×90°	−4.75/−1.50×180°	−2.75×180°	+3.25×90°
Axial length (mm, OD/OS)	21.47/21.28	20.30/19.85	NA	ND	ND	ND	ND	ND	ND
Fundus features	NA	NA	NA	Mid-peripheral pigment alterations, vessel attenuation	A pale fundus, mid-peripheral pigment alterations, vessel attenuation	A pale fundus, diffuse atrophic changes of the retinal pigment epithelium, vessel attenuation, bone spicule-like pigmentation	A blurring pale fundus, mid-peripheral pigment alterations, vessel attenuation	A pale fundus, mid-peripheral pigment alterations, vessel attenuation, bone spicule-like pigmentation	Slight mid-peripheral pigment alterations, slight vessel attenuation
Cataract (OD/OS)	Severe/Severe	Severe/Severe	Severe/Severe	No/Severe	Moderate/Severe	No/Moderate	Severe/Severe	Moderate/No	No/No
Cataract subtype (OD/OS)	TC/TC	TC/TC	TC/TC	No/PSC	CC/CC	No/PSC	PSC/PSC	PSC/No	No/No
OCT (CFT, μm) (OD/OS)	ND	ND	ND	ND/145	EM (310/262)	160/155	190/168	107/152	209/225

Abbreviations: BCVA, best corrected visual acuity; CC, cortical cataract; CFT, central foveal thickness; EM, epiretinal membrane; F, female; LP, light perception; M, male; NA, not available; ND, not detected; NLP, no light perception; OCT, optical coherence tomography; OD, right eye; OS, left eye; PSC, posterior subcapsular cataract; *RHO*, the rhodopsin gene; TC, total cataract.

### Whole exome sequencing

Proband (III:4, Figure 1A) WES showed that ∼44.04 million reads (99.6%) mapped to the human reference genome. Average sequencing depth on the target region was 72.65. A large proportion of the region, 98.7%, was covered by the target sequence at 10× or greater. A total of 121690 SNPs were detected, including 22046 in the exon regions and 2399 in the splicing sites. Testing identified 13489 insertions/deletions, including 562 in the exon regions, and 379 in the splicing sites.

### Bioinformatics analysis and *RHO* mutation screening

A prioritization program was applied to identify any proband pathogenic variants. Public databases, including dbSNP137, 1000 genomes project and NHLBI ESP6500 were used to filter commonly known variants. In the proband, only one heterozygous variant, c.403C>T (p.R135W), of the *RHO* gene exon 3 was suspected to be a pathogenic variant. No other variants in known disease-causing genes for vision degeneration-related disease were identified. Sanger sequencing confirmed the heterozygous variant present in all affected family members (II:3, II:5, II:7, III:1, III:2, III:4, III:6, III:11 and IV:1) (Figure 1B), but absent in five unaffected family members (III:7, III:12, IV:2, IV:3 and IV:4). The variant co-segregates with the disease in family members and was absent in the 100 unrelated Chinese healthy controls (Figure 1C). This suggests that the variant may be the pathogenic mutation in this family.

Arginine at position 135 (p.R135) is conserved across different species (Figure 1D). The SIFT analysis of mutation obtained a score of 0.00, which suggests a damaging mutation. PolyPhen-2 prediction showed the mutation as “probably damaging” with a score of 1.00 on HumVar (sensitivity, 0.00; specificity, 1.00). MutationTaster analysis indicated that the substitution was disease-causing with a high-confidence probability value close to 1.

### Subcellular location prediction

Based on the TMHMM prediction, wild-type p.R135 locates at the second rhodopsin CP loop (amino acids from 134 to 152), but the mutated p.W135 locates at the third TM helice (amino acids from 116 to 138).

## Discussion

The *RHO* gene, located at chromosome 3q22.1, contains five exons. It encodes a 348-amino acid rod-specific protein rhodopsin which is a typical seven TM G-protein-coupled receptor [22]. When rhodopsin absorbs the photon, the retinal chromophore (11-*cis*-retinal) changes to an all-trans-retinal, leads to a phototransduction cascade activation, which plays an important role in vision [11,23].

Since the first p.P23H mutation in the *RHO* gene was discovered in 1990 [24], more than 210 different mutations, which vary from point mutations to complex rearrangements, have been reported in different ethnic populations (HGMD Professional, http://www.hgmd.cf.ac.uk/ac/index.php).

Most *RHO* mutations cause adRP. However, homozygous *RHO* mutations p.E150K, p.W161X, p.E249X and IVS4+1G>T are reported in a few autosomal recessive RP families [22,25–30]. Heterozygous mutations (p.G90D, p.T94I, p.A295V and p.A292E) are found in patients with congenital stationary night blindness [31].

RP is exceptionally heterogeneous, genetically, allelically, phenotypically and clinically [32]. *RHO* mutations have been reported to be phenotypically heterogeneous in two instances: classic RP and sector RP. Classic RP is a typical RP characterized by early onset and diffuse or generalized retinal dysfunction. Sector RP is characterized by adult onset and regionalized or sectorial retinal dysfunction [11]. At least 13 *RHO* gene mutations associate with sector RP [11,23,33–35].

**Figure 4:**
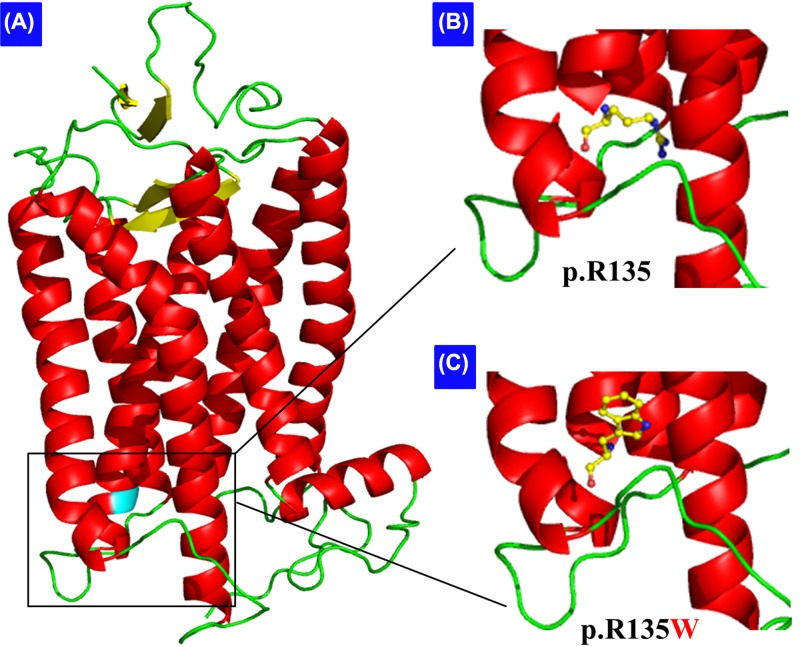
Cartoon representation of the model structure of the rhodopsin protein (**A**) Overall structure of the protein is displayed in three dimensions. (**B**,**C**) Amino acids at position 135, the arginine (B) and the mutated tryptophan (C), are shown as ball-and-stick models.

Our study found a reported *RHO* p.R135W mutation, which has also been found in other ethnicities, including Han-Chinese, Korean, Indian, Turkmenistan Jews, North African Jews, Caucasian, Swedish, French and others (Table 2) [2,3,10,36–40]. p.R135 locates on the border between the second CP loop and the third TM domain of rhodopsin [41]. The chain near position 135 may play a critical function in rhodopsin folding. In addition, R135 creates a salt-bridge with E134 and E247 adjacent to TM helices III and VI, respectively, forming an ionic lock which holds helices III and VI together at their CP ends while keeping the receptor as inactive state [42]. The p.R135W mutation may disrupt ionic interactions and result in partial misfolding and a reduced mutant protein ability to bind 11-*cis*-retinal (Figure 4) [40,42]. p.R135 seems to be a ‘hot’ mutational site with several other mutations being reported including p.R135P, p.R135L, p.R135G and p.R135W [2,3,10,36–40,43–45]. The p.R135-based mutants can be phosphorylated by rhodopsin kinase and bind arrestin, but cannot activate transducin, in the absence of 11-*cis*-retinal [41]. Among these mutations, p.R135W leads a more seriously and rapidly progressive RP than p.R135L, as its glycosylation state is more defective [38]. *In vitro*, bovine p.R135W rhodopsin mutants disrupt G protein activation, contrary to the wild-type rhodopsin [40,41].

**Table 2:** p.R135W mutation detected in the *RHO* gene in patients with RP

Report	Geographic and ethnic group	Detection method	Family/sporadic	Inheritance pattern	Clinical features in the patients^*^
Sung et al., 1991 [36]	Caucasian	DGGE	Family	AD	(1) (3)
Bareil et al., 1999 [37]	French	DGGE	Family	AD	(2) (3) (4) (5)
Iannaccone et al., 2006 [38]	Indian	Direct sequencing	Family	AD	(2) (3) (4) (5)
Kim et al., 2012 [2]	Korean	Direct sequencing	Family	AD	(2) (3) (4) (6) (7)
Wu et al., 2014 [3]	Chinese	WES	Family	AD	(1) (2) (3) (4) (6)
Beryozkin et al., 2016 [10]	North African Jews	Sanger sequencing	Family	AD	(2) (4)
Beryozkin et al., 2016 [10]	Turkmenistan Jews	Sanger sequencing	Family	AD	(2) (4)
Abdulridha-Aboud et al., 2016 [39]	Swedish	Genotypic microarray	Family	AD	(2) (3) (4) (5) (6)
Yu et al., 2016 [40]	Chinese	CNGS	Family	AD	(1) (2) (4) (5) (6) (9) (10)
The present study	Chinese	WES	Family	AD	(1) (2) (3) (4) (5) (6) (7) (8) (9) (10) (11)

* Clinical features in the patients: (1) night blindness; (2) reduced visual acuity; (3) reduced peripheral visual field; (4) typical fundus findings; (5) abnormal electroretinography result; (6) abnormal OCT result; (7) cataract; (8) hyperopic refractive errors; (9) myopic refractive errors; (10) astigmatism; (11) epiretinal membrane.Abbreviations: AD, autosomal dominant; CNGS, capturing next-generation sequencing; DGGE, denaturing gradient gel electrophoresis; *RHO*, the rhodopsin gene; WES, whole exome sequencing.

High clinical heterogeneity and distinct features were present in this family. All affected patients began having severe night blindness early in life, consisting with other families with *RHO* p.R135W mutation [3]. Nearly half, 44.4% (4/9), of the patients (II:3, II:5, III:2 and IV:1) have hyperopic refractive errors, a notably higher percentage than most reported RP patients, of whom 75–77.5% have myopia, and only 7.6% have hyperopia [4,6]. Eight of nine patients present with cataracts either unilaterally, or bilaterally, 72.2% (13/18) eyes. This is nearly twice the previously reported morbidity of ∼45% [4,5]. Early cataracts may associate with mild inflammatory reactions [46]. This observation is supported by the fact that transgenic mice with rhodopsin promoter-induced interferon-gamma (IFN-γ) expression tend to develop cataract formation [47]. Patient (III:2) has bilateral EM, and EM is present in 8.2–27.3% of the eyes of RP sufferers in recent reports [48,49]. The complex clinical manifestations observed in this family may result from many factors including genetic background, environmental effects and epigenetic modifications.

## Conclusions

An *RHO* gene missense mutation, c.403C>T (p.R135W) was identified in a Han-Chinese family with adRP. The present study extends the genotype–phenotype relationship between the *RHO* gene p.R135W mutation and adRP clinical findings. These results have implications for family genetic counseling, clinical management and developing RP target gene therapy strategies.

## Abbreviations

adRP: autosomal dominant RP
BCVA: best corrected visual acuity
CP: cytoplasmic
EM: epiretinal membrane
ERG: electroretinography
gDNA: genomic DNA
IFN-γ: interferon-gamma
*IMPDH1*: the inosine monophosphate dehydrogenase 1 gene
logMAR: logarithm of the minimum angle of resolution
*NR2E3*: the nuclear receptor subfamily 2 group E member 3 gene
OCT: optical coherence tomography
PolyPhen-2: Polymorphism Phenotyping version 2
*PRPF8*: the pre-mRNA processing factor 8 gene
*PRPF31*: the pre-mRNA processing factor 31 gene
*PRPH2*: the peripherin 2 gene
*RHO*: the rhodopsin gene
RP: retinitis pigmentosa
*RP1*: the RP1 axonemal microtubule associated gene
*RPGR*: the RP GTPase regulator gene
SIFT: Sorting Intolerant from Tolerant
SNP: single nucleotide polymorphism
TM: transmembrane
TMHMM: TransMembrane prediction using Hidden Markov Model
WES: whole exome sequencing
